# Budgeting for comprehensive sexual and reproductive health and rights under universal health coverage

**DOI:** 10.1080/26410397.2020.1779631

**Published:** 2020-07-27

**Authors:** Naomi Lince-Deroche, Elizabeth A Sully, Lauren Firestein, Taylor Riley

**Affiliations:** a Senior Research Scientist, Guttmacher Institute, New York, NY, USA.; b Research Associate, Guttmacher Institute, New York, NY, USA; c Senior Research Associate, Guttmacher Institute, New York, NY, USA

**Keywords:** sexual and reproductive health, reproductive rights, universal health coverage, costs, budgeting

## Abstract

Achieving universal health coverage (UHC) for sexual and reproductive health (SRH) requires informed budgeting that is aligned with UHC objectives. We draw data from *Adding It Up 2019 (AIU-2019)* to provide critical new country-level and regional, intervention-specific costs for the provision of SRH services. AIU-2019 is a cost-outcomes analysis, undertaken from the health system perspective, which estimates the costs and impacts of offering SRH care in low- and middle-income countries. We present direct cost estimates for 109 SRH interventions and find that human resources comprise the largest category of direct SRH service costs and that the most expensive services in the model are largely preventable. We use scenario analysis to explore the synergistic costs and impacts of providing SRH interventions in clusters, focussing on chlamydia and gonorrhoea treatment, provision of safe abortion and post-abortion care services, and safe childbirth services. When costs are considered for the preventive and impacted services in these three clusters, there are cost savings for some of the impacted services in the packages and for the abortion-related package overall. The direct cost estimates from our analysis can be used to guide UHC budgeting and planning efforts. Having these cost estimates and understanding the potential for cost savings when providing comprehensive SRH services are critical for efforts to fulfil the rights and needs of all individuals, including the most marginalised, to access this essential care.

## Résumé

Une couverture santé universelle (CSU) pour la santé sexuelle et reproductive (SSR) requiert une budgétisation informée qui soit alignée sur les objectifs de la CSU. Nous avons extrait des données du rapport Adding It Up 2019 (AIU-2019) pour fournir de nouveaux coûts essentiels, spécifiques aux interventions et de niveau national et régional pour la prestation de services de SSR. Le rapport AIU-2019 est une analyse coût-résultat, entreprise dans la perspective des systèmes de santé, qui estime les coûts et les impacts de l’offre de soins de SSR dans des pays à revenu faible ou intermédiaire. Nous présentons des estimations du coût direct de 109 interventions de SSR et constatons que les ressources humaines représentent la principale catégorie de coût direct des services de SSR, et que les services les plus onéreux dans le modèle peuvent être en grande mesure prévenus. Nous utilisons alors une analyse des différents scénarios pour étudier les coûts synergétiques et l’impact de l’offre d’interventions de SSR dans des groupes. Nous nous centrons sur les traitements de la gonorrhée et la chlamydia, la prestation de services d’avortement sans risque et de soins post-avortement ainsi que de services obstétricaux sûrs. Quand les coûts sont examinés pour les services préventifs et impactés dans ces trois groupes, on observe des économies de coût pour certains des services impactés dans le panier et pour l’ensemble des prestations dans le panier relatif à l’avortement. Les estimations des coûts directs émanant de notre analyse peuvent permettre de guider les activités de budgétisation et de planification de la CSU. Il est essentiel de disposer de ces estimations de coût et de comprendre le potentiel de réductions de coût quand on assure des services complets de SSR pour que tous les individus, y compris les plus marginalisés, puissent jouir du droit de bénéficier de ces soins essentiels et satisfaire leurs besoins dans ce domaine.

## Resumen

Para lograr la cobertura universal de salud (CUS) para la salud sexual y reproductiva (SSR) se necesita una presupuestación informada en consonancia con los objetivos de CUS. Extraímos datos de *Adding It Up 2019 (AIU-2019)*, para proporcionar nuevos costes a nivel nacional y regional, relacionados con intervenciones específicas, que son fundamentales para la prestación de servicios de SSR. AIU-2019 es un análisis de resultados de costes, realizado desde la perspectiva del sistema de salud, que calcula los costes e impactos de ofrecer servicios de SSR en países de bajos y medianos ingresos (PBMI). Presentamos estimaciones de costes directos para 109 intervenciones de SSR, y encontramos que los recursos humanos constituyen la mayor categoría de costes directos de los servicios de SSR, y que los servicios más costosos del modelo son en gran parte evitables. Utilizamos el análisis de escenarios para explorar los costes e impactos sinérgicos de proporcionar intervenciones de SSR en agrupaciones. Nos enfocamos en el tratamiento de clamidia y gonorrea, en la prestación de servicios de aborto seguro y de atención postaborto, y en los servicios de parto seguro. Cuando se consideran los costes de los servicios preventivos e impactados en estas tres agrupaciones, hay ahorros de costes para algunos de los servicios impactados en los paquetes y para el paquete de servicios relacionados con el aborto en general. Las estimaciones de costes directos calculados en nuestro análisis pueden utilizarse para guiar los esfuerzos de presupuestación y planificación de CUS. Tener estas estimaciones de costes y entender el potencial de ahorrar costes durante la prestación de servicios integrales de SSR son factores críticos para los esfuerzos por cumplir los derechos y las necesidades de todas las personas, incluidas las más marginadas, de acceder a estos servicios esenciales.

## Introduction

The principle of “health for all” has been a driving force behind progress in health care for centuries^1,2^ Its newest incarnation, now called universal health coverage (UHC), is defined by the World Health Organization (WHO) as a state where all people can access the quality health services they need without experiencing financial hardship.^3^ Support for UHC is widespread: a global commitment was solidified at the United Nations General Assembly (UNGA) in 2012.^4,5^ The principle was adopted as part of the sustainable development goals (SDGs) in 2015,^6^ and in 2019, UNGA held the first high-level meeting on UHC reaffirming the right of all human beings to the “highest attainable standard of physical and mental health.”^7^

While some countries have arguably achieved UHC already, many more are making progress. There are two key questions that any country seeking to achieve UHC must address: how to finance UHC and how to organise the health system to ensure optimal service delivery.^8^ Answering these questions in the context of limited resources obligates countries to undertake priority setting or determining which services should be funded within the schemes available. Because many countries simply cannot afford to offer access to comprehensive care for all immediately, priority setting speaks to the need for the progressive realisation of universal access to care. Fortunately, mechanisms for priority setting in health already exist in many settings. Unfortunately, politics often play a significant role^9^ and, coupled with a lack of transparency, can potentially put services for historically marginalised populations (e.g. youth) or stigmatised services (e.g. safe abortion) at risk of being sidelined.

Political interference and obfuscation of decision-making processes for the recognition and provision of sexual and reproductive health and rights (SRHR) have been a reality in the international arena for some time. However, a recent international call for renewed action on SRHR, the Guttmacher-*Lancet* Commission report, puts forward a comprehensive definition of SRHR and calls for investment in a package of SRH interventions that fully addresses the rights and needs of all individuals, including with respect to chronically neglected issues such as violence, stigma, and bodily autonomy.^10^ The report also acknowledges that meeting all need for SRH care may be affordable for all but the poorest countries and that investing in these services would engender significant health, financial, and social benefits. While the report highlights the cost-effectiveness of key SRH care, like contraceptive and maternal and newborn health services, in many settings, it also highlights the lack of data on the costs of offering many other types of recommended SRH services.

There is growing recognition that no amount of increased revenue for UHC will ensure equitable and efficient service delivery if systems for financial management, including budgeting, are not aligned with programmatic objectives.^11^ Budgeting for a select few, rather than the comprehensive set of SRH services that individuals require, will lead to falling short of the rights-based aims of the UHC discourse. As such, it is critical for domestic policymakers and other stakeholders to have information on the costs of offering SRH services and an understanding of the potential financial impacts of these investments.

Several international efforts have attempted to address this need, estimating the cost implications of providing various SRH services.^12–17^ Since 2003, the Guttmacher Institute has produced *Adding It Up*, a report on the costs and impacts of meeting all need for modern contraceptive services, pregnancy-related and newborn health care, and treatment for four curable sexually transmitted infections (STIs).^18–23^ *Adding It Up* has demonstrated the advantages of simultaneous investments in SRH services, showing that meeting all need for both modern contraception and pregnancy-related and newborn care generates greater health impacts than investing in either one alone and also produces overall cost savings in terms of the investment required to obtain the estimated impact.^23^

In this paper, we take advantage of data from *Adding It Up 2019* (AIU-2019) to provide critical new country-level and regional, intervention-specific costs for the provision of SRH services. These data can be used to bolster efforts to include essential SRH services in UHC budgeting efforts. We also present total costs for providing groups of SRH services – as individual services and when offered together – in order to underscore the interrelated nature and synergistic impact of investing in SRH as a package of care rather than as individual services. Having these cost estimates and understanding the potential for cost savings when providing essential SRH services are critical for efforts to fulfil the rights and needs of all individuals, including the most marginalised, to access this essential care.

## Material and methods

### The AIU model

All data for this analysis are drawn from AIU-2019, which is a cost-outcomes analysis, undertaken from the health system perspective.^23^ Detail on the AIU-2019 methodology can be found elsewhere.^24^ In summary, the analysis employs bottom-up costing methodology and includes extensive compilation and computation of country-level data on demographics, service needs and utilisation, recommended standards of care, and the health system cost of providing that care.

The AIU-2019 model produces one-year estimates of the costs and outcomes, or health impacts, from the provision of SRH services to women of reproductive age and their newborns in all low- and middle-income countries (LMICs), as defined by the World Bank.^25^ The model includes all treatment interventions deemed essential for the provision of modern contraception, pregnancy-related care (including abortion), newborn care and treatment of four curable STIs based on guidance from WHO and other international sources or experts (See Supplementary Table 1). In this paper, we focus on the direct costs of the 109 costed interventions in AIU-2019. Information on the impacts of providing the interventions and the indirect cost of offering care can be found elsewhere.^23^

### Costing approach

We estimated direct costs for each intervention included in the AIU-2019 model by first establishing the “ingredients” required for care within four categories, and then applying the costs for each ingredient and summing to produce a total direct cost per category and per intervention. The direct cost categories included drugs and supplies, contraceptive commodities, personnel, and in-hospital food costs.

In preparing the list of required cost ingredients, we considered all elements of care that should be provided. We drew from WHO policies and guidelines, Cochrane Reviews, peer-reviewed clinical and costing literature, and in cases with no published data or guidance, we relied on expert opinion. We included variations that might be required for varying disease severity or prevalence. These variations were incorporated through the use of proportional weights. Table 1 provides an example of the ingredients list and weights used for routine vaginal delivery. It illustrates the assumption that half of the women receiving this intervention will have an episiotomy. It also indicates that the average patient may be monitored by a nurse/midwife or assistant nurse during delivery. A full list of the ingredients for every intervention included in the AIU-2019 model can be found online as part of the detailed methodology report.^24^ Note that costs thought to incur exclusively to women or otherwise outside of the health system were not included in the AIU-2019 cost model. For example, the cost of drugs or supplies procured by women to self-induce an abortion were excluded, as were the costs of time spent by individuals to clandestinely provide unsafe abortion services.

**Table 1.** Ingredients example: essential care for all women with routine vaginal delivery

Drug/supply	% receiving	Note	No.	Times per day	Days per case	Total units per case	Unit cost (any country)	Cost per avg. case (any country)
Drawsheet, plastic	100	Assumes cleaned and reused at least 2 times*	0.3	1	1	0.3	$0.84	$0.28
Gloves, latex, disposable, pair	100	One per health care worker*	1	4	1	4	$0.05	$0.19
Chlorhexidine surgical scrub, 5ml	100	Antiseptic*	1	1	1	1	$0.02	$0.02
Cotton swab	100	For application of antiseptic^†^	1	1	1	1	$0.10	$0.10
Partograph	100	For monitoring*	1	1	1	1	$0.04	$0.04
Delivery record	100	For documenting*	1	1	1	1	$0.18	$0.18
Identification bracelet	100	For the woman^†^	1	1	1	1	$0.03	$0.03
Paracetamol, tablet, 500 mg	100	For pain relief*	1	4	3	12	$0.01	$0.11
*Episiotomy*								
Lidocaine HCl (in dextrose 7.5%), ampoule 2ml	50^ǂ^	Local anaesthesia for episiotomy /repair of tears*	1	1	1	1	$0.13	$0.06
Syringe, needle and swab	50^ǂ^	For lidocaine injection*	1	1	1	1	$0.07	$0.04
Suture, catgut, chromic, needle	50^ǂ^	Suture of episiotomy or tear*	1	1	1	1	$0.29	$0.15
Gauze pad, sterile	50^ǂ^	Dressing for episiotomy/tears*	1	1	1	1	$0.06	$0.03
Staff Type	% treated by			Min’s^†^	No. of days	Total min’s	Unit cost (example: Bangladesh)	Cost per avg. case (example: Bangladesh)
Nurse/Midwife	50^§^	Monitoring of labour, assistance during delivery*		120	1	120	$0.06	$3.75
Assistant Nurse	50	Monitoring of labour, assistance during delivery^§^		120	1	120	$0.05	$2.88
Obstetrician	100	During delivery*		20	1	20	$0.08	$1.54
Auxiliary/ Attendant	100	During hospital stay^§^		30	2	60	$0.05	$2.88
Hospital food cost	% hospitalised				No. of days	Total days	Unit cost (example: Bangladesh)	Cost per avg. case (example: Bangladesh)
Food costs per day	100*				2		$0.56	$1.12

No., number; Min’s, minutes; Avg., average.

* These assumptions follow those included in the OneHealth Tool.^46^. Note that some delivery-related items included in OneHealth are included as essential care for newborns in the *Adding It Up* model.

^†^Assumptions based on advice from clinicians and health system costing experts.

^ǂ^Assumptions based on information contained in Clesse et al.^56^

^§^Assumptions based on the WHO’s guidance for task-shifting for maternal and child health.^49^

### Data

#### Drugs and supplies

We obtained prices for almost all the roughly 145 medications, diagnostic tests and consumable supplies in the AIU-2019 model from internationally recognised suppliers. More than 60% of the prices came from UNICEF;^26^ others were drawn from Management Sciences for Health’s International Medical Products Price Guide,^27^ the IDA Foundation,^28^ CHAI,^29^ The Global Fund,^30^ and UNFPA.^31^ Finally, a small number of prices were obtained from a wholesale online retailer^32^ or single-country price catalogues.^33–35^ Prices for oxygen and blood products were extremely difficult to find, and we thus made assumptions based on online sources^36–38^ and expert advice. Because nearly all prices were obtained from global sources, once obtained, we assumed all prices were the same across all countries in the model.

Most prices for drugs and supplies were reported by the sources noted above for 2018; some items had older prices. Based on a historical review of product catalogues from the sources supplying the majority of the required prices for the *Adding It Up* analysis, we decided to assume stable prices over short periods (in this case, from 2015 forward), so we did not inflate prices given for 2015 or more recent years. A few additional items had prices older than 2015. For those, we inflated to 2019 values using Gross Domestic Product (GDP) deflators.^39^ We capped year-on-year inflation at 200% in countries that had experienced very high or hyperinflation over the period of interest. Inflation rates for countries missing deflator information (i.e. Palestine and North Korea) were imputed based on average annual inflation from within the country’s UN Population Division subregion.^40^ Finally, we increased all the drug and supply prices to account for shipping (15%) and wastage (30%) costs. Wastage was assumed to include expiry, damage, and other losses prior to dispensing.

#### Contraceptive commodities

For AIU-2019, we utilised contraceptive price information provided by the Reproductive Health Interchange (RHI) database, which includes price data for contraceptive orders and shipments.^41^ We used data for a four-year period (1 January 2015–31 December 2018) to calculate a weighted, per method unit cost for each country using the quantity and price of every shipment to a given country. We did not inflate or add shipping and wastage costs because the RHI cost data include the commodity’s unit price, shipping, insurance, and any related fees. For countries with missing method price data, we imputed the unweighted average cost in the country’s geographic subregion.^42^ Missing price data were more common for less commonly used methods, like the cycle beads used for fertility awareness-based method costs, where just 8% (11/132) of countries had price data, and for the emergency contraceptive pill (available for 49% (65/132) of countries). More commonly used methods were more likely to have price data in the RHI database (e.g. implants – 61%, IUDs – 70%, pills – 70%, injectables – 73%, and male condoms – 85% of countries) (data not shown).

Then, we annualised contraceptive method unit costs to reflect couple-years of protection (CYPs) (i.e. estimates of the number of commodity units required to provide one year of contraceptive protection). We generally followed accepted guidelines for annualisation.^24,43^ In summary, for short-term methods we multiplied the commodity unit costs by a conversion factor representing the number of units required to provide one year of protection. For long-term, reversible methods (e.g. IUD or implant) and sterilisation, we divided the method cost by a conversion factor representing the average number of years of protection expected. More information on the conversion factors and our methodology can be found elsewhere.^24^

For methods with more than one product type available (e.g. implants, contraceptive pills), we collapsed product-specific annualised costs to create weighted average method category costs. The weights used reflect the country-specific proportional representation of the method types in the shipments recorded in the RHI database. Finally, some geographic subregions lacked contraceptive commodity cost data. For countries in those subregions, we imputed average annualised costs from another suitable subregion, as explained elsewhere.^24^

#### Personnel

For personnel costs, we used country-specific salaries for four categories of health care workers as published by WHO’s Choosing Interventions that are Cost Effective (WHO-CHOICE) initiative.^44^ The salaries include the salary, paid vacation, and other regularly included items (such as social security, health insurance and bonuses). For AIU-2019, we inflated the salaries to 2019 (following the methodology noted above for drugs and supplies) and mapped the four WHO-CHOICE categories onto the 12 categories of health professionals required to offer the interventions included in the model using methodology described separately.^24^ For countries not included in the WHO-CHOICE data (7/132 (0.05%)), we used unweighted averages from countries with data in the same WHO subregion.^45^ Finally, we converted annual salaries to costs-per-minute assuming 48 weeks of work per year and 30 hours of work per week following the assumptions used in OneHealth, another widely recognised costing tool.^46^

#### In-hospital food provision

In prior iterations of *Adding It Up*, daily food costs were assumed to be US$0.50 per person per day for food in all LMICs.^47^ In AIU-2019, this point estimate was stratified using the proportional distribution of GDP per capita in each country as described in detail elsewhere.^24^

### Analysis

#### All interventions – average direct costs

Budgeting for UHC requires information on the costs of offering care to individuals in need. We present the total direct health system cost of providing each SRH intervention to an average recipient as described above. These costs are presented for all LMICs in Supplementary Table 1. We also provide the average regional direct cost per intervention when broken down by cost category (i.e. drugs and supplies, contraceptive commodities, personnel, and in-hospital food provision) in Supplementary Table 2. The regional category costs are weighted using the proportional representation of women aged 15–49 in each country in the region.

#### Interlinked intervention examples – scenario analysis

Budgeting for UHC also requires an understanding of the cost implications of offering services as a package of care to all who require the various services. AIU-2019 addresses this need using a scenario analysis approach, including scenarios for (1) current levels of care and (2) all-needs-met – where all needs for services are satisfied. In each scenario, total direct costs for all individuals receiving care are calculated by multiplying the average direct cost per intervention by the number of women or newborns in need and receiving care. The methodology for determining the number of women or newborns in need and receiving care has been described separately.^24^ It is intervention-specific and involves using country-level population health care coverage data from a variety of sources.

For this paper, we again used scenario analysis to explore three examples of how meeting all need for one preventive service within a cluster of interlinked services impacts need and costs. The three preventive service examples we included were: (1) treatment of chlamydia and gonorrhoea, (2) provision of safe abortion, and (3) safe childbirth services (i.e. essential care for all women during delivery and for their newborns). These three clusters include 31 of the 109 total costed interventions in the model. They were selected for their ability to illustrate the impact of investing in one or more services on other related services without complex intermediary steps. We present the cost of providing these clusters, which include services and the services that they impact, by region and in all LMICs for three scenarios. These include (1) current levels of care for all services in the cluster, (2) current care for the preventive service and all-needs-met for the impacted service(s), and (3) all-needs-met for the preventive and impacted services. Table 2 lists the preventive and impacted services in each interlinked cluster as well as the parameters and assumptions employed in each scenario. Table 3 provides the number of women in 2019 who were aged 15–49, living in all LMICs, and receiving the interventions in each interlinked cluster. (Supplementary Table 3 provides these numbers by region.) Below is the additional detail on the services and the linkages between them.

**Table 2.** Interlinked services and analysis parameters by scenario

Preventive service(s)	Impacted service(s)	Assumptions/mechanism by scenario		
		(1) Current service levels	(2) All-needs-met: Impacted service(s)	(3) All-needs-met: Preventive & impacted services
Chlamydia (CT) and gonorrhoea (NG) treatment	Pelvic inflammatory disease (PID) treatment	Level of treatment is country-specific. However, many women with CT and NG go untreated, 40% of untreated women progress to PID, ^57^ and many of these PID cases go untreated	Level of treatment is country-specific. Women with CT and NG go untreated, 40% of untreated women progress to PID,^57^ and all of these PID are treated	All incident CT and NG infections are treated, eliminating the PID that results from untreated infections
Safe abortion services	Unsafe abortion* and postabortion care (PAC)	Levels of safe and unsafe abortion services are country-specific. We used estimates from country-specific studies, prior AIU estimates, and abortion safety classifications to make assumptions about estimated abortion complications that require PAC. More details can be found elsewhere.^24^	Levels of safe and unsafe safe services remain at current country-specific levels. All need for abortion-related PAC is met.	We hypothetically assumed that all abortions are provided safely. Need for abortion-related PAC is greatly reduced.^†^ All PAC cases receive treatment.
Safe childbirth services^ǂ^	Services for managing/treating: - Maternal sepsis - Post-partum haemorrhage (PPH) - Obstetric fistula - Local infections in newborns - Newborn sepsis	The level of facility-based, safe deliveries is country-specific. The level of care provided for the following complications requiring treatment is also country-specific: 2.5% of vaginal deliveries in facilities, 5.3% of caesarean deliveries and 5% of deliveries outside of facilities progress to maternal sepsis.^58^ Among women delivering vaginally, including assisted vaginal deliveries (AVDs), among those receiving active management of third-stage labour (AMSTL), 9.5% experience post-partum haemorrhage (PPH). Among women delivering vaginally who do not receive AMSTL, PPH occurs at a rate of 21.9%.^59^ 2.15% of women with untreated obstructed labour experience obstructed fistula 10% of newborns experience local infections,^60^, and 10% of newborns develop sepsis.^61^ 90% of newborns with sepsis can be treated with injectable antibiotics, and 10% require full supportive care.^62^	Facility-based, safe deliveries remain at current country-specific levels. However, care for complications a-d increases to meet all need	We assume all women giving birth have facility-based, safe delivery care. We also assume all newborns are delivered with safe, clean delivery practices and all receive immediate newborn care. As a result, complications drop as noted below. All complications receive treatment. All women undergoing caesarean section receive prophylactic antibiotics, reducing maternal sepsis to 2.9% of caesarean deliveries and 2.5% of vaginal deliveries in facilities.^58^ All women delivering vaginally receive AMSTL, reducing PPH to 9.5% of these deliveries. We assume all obstetric fistula is eliminated due to all cases of obstructed labour receiving treatment. Occurrence of newborn infections and newborn sepsis is reduced by 50%.^60^ The sepsis treatment needs (injection or full care) remain split at 90%/10%

CP, contraception; PRNC, pregnancy-related and newborn care; CT, *Chlamydia trachomatis* (chlamydia); NG, *Neisseria gonorrhoeae* (gonorrhoea) PID, pelvic inflammatory disease.

*Unsafe abortion comprises both less safe and least safe abortion.

^†^A low level of need for PAC persists due to complications from safe abortion.

^ǂ^ Essential care for all women with routine vaginal delivery, assisted vaginal delivery or caesarean section plus essential care for all newborns

**Table 3.** Number (000s) or proportion of women aged 15–49 requiring and receiving service in all LMICs in 2019, by scenario*

Scenario:	(1) Current service levels	(2) All-needs-met: Impacted service(s)	(3) All-needs-met: Preventive & impacted services
**Cluster 1: STI treatment for women aged 15–49**			
Number of women with chlamydia and gonorrhoea infections	87,194	87,194	87,194
Number of women who receive chlamydia and gonorrhoea treatment	10,986	10,986	87,194
% of all chlamydia and gonorrhoea infections that go untreated	87%	87%	0%
Number of women with PID	30,483	30,483	0
Number of women with PID who receive treatment	16,285	30,483	0
% of PID infections that go untreated	47%	0%	NA
**Cluster 2: Induced abortion and postabortion care**			
Number of safe abortions	33,099	33,099	68,518
Number of less safe abortions	24,268	24,268	0
Number of least safe abortions	11,151	11,151	0
Number of women requiring PAC^†^	20,882	20,882	1,655
Number of women receiving PAC^†^	12,340	20,882	1,655
% of women who need but do not receive PAC^†^	41%	0%	0%
**Cluster 3: Safe childbirth services**			
Number of live births in LMICs	127,362		127,362
*Preventive*			
Number of births with facility deliveries	96,644		127,362
Number of newborns receiving immediate care	96,644		127,362
*Impacted*			
Obstetric fistula repair^ǂ^			
Number needing	68		0
Number receiving	22		0
Maternal sepsis management^ǂ^			
Number needing	4,673		3,251
Number receiving	2,811		3,251
Postpartum haemorrhage treatment^ǂ^			
Number needing	13,274		7,939
Number receiving	6,524		7,939
Newborn local infections treatment			
Number needing	12,736		6,368
Number receiving	9,664		6,368
Newborn sepsis treatment (injectable antibiotics and full supportive care)			
Number needing	12,736		6,368
Number receiving	9,489		6,368

LMIC, low- and middle-income country; PID, pelvic inflammatory disease; NA, not applicable; PAC, postabortion care.

*Regional outcomes can be found in Supplementary Table 3.

^†^For abortion complications (i.e. not including PAC for miscarriage).

^ǂ^These costs are only for women needing these interventions who have live births. Those needing these interventions who have a stillbirth are excluded here.

The first interlinked service cluster focuses on the treatment of two of the most common, curable STIs globally: chlamydia and gonorrhoea. In the current coverage scenario, some treatment is provided, but many women go untreated. We assume that a portion of untreated cases progress to pelvic inflammatory disease (PID) and present total costs for treatment of chlamydia, gonorrhoea and PID at current levels. In the all-needs-met scenarios, we first present total costs when treatment of incident chlamydia and gonorrhoea infections remain at current levels, but all chlamydia- or gonorrhoea-induced PID is treated. Then we present total costs when incident chlamydia and gonorrhoea infections are treated and the need for PID treatment falls away.

For the second example, abortion services, the current care scenario includes coverage at current levels. Following a recently established definition for representing abortion services as safe, less safe, and least safe,^48^ we include costs for providing current levels of safe abortion services, i.e. dilation and evacuation, vacuum aspiration (VA), and medical abortion (MA). We also include health system costs for less safe procedures. These include costs for the provision of dilation and curettage, which is not recommended by the WHO, the cost of providing VA with inadequate staffing, and MA drug costs only (assuming no health system costs are incurred for personnel to provide the drugs). “Least safe” services are assumed to be provided outside of the health system, or self-induced, and as a result, no costs are included. Finally, in the current coverage scenario, we included all costs for providing post-abortion care (PAC) for abortion-related complications. For the all-needs-met scenarios, we first assumed that abortions would continue to happen under current, country-specific conditions, but that all need for PAC would be met. Then, we assumed that all abortions would be provided in safe conditions (requiring hypothetical liberalisation of laws and full access to care). Some need for PAC would persist due to low levels of complications from safe abortion. We assumed that all need for this PAC would be met.

In our third example, we focus on the provision of safe childbirth services, i.e. essential safe delivery care for women and essential care for newborns, and the impact of offering this care on maternal and neonatal outcomes around the time of delivery. Because childbirth can result in complications even when occurring under high-quality conditions, the relationship between the need for and impact of offering the many interventions that comprise the childbirth-related cluster in the full AIU-2019 analysis is highly complex. For this analysis, we focus on a smaller cluster of interventions representing select, immediate impacts from low-quality delivery services and newborn care. These include obstetric fistula repair, maternal sepsis case management, and postpartum hemorrhage treatment for mothers and management of infections and sepsis in newborns. The current care scenario assumes current levels of delivery-related care and complication management. Due to the structure of the AIU-2019 model, there is no intermediate step to present in the first all-needs-met scenario, where one preventive service can be held constant. As a result, we present need and costs for the second all-needs-met scenario only. There, we assume that all need for in-facility deliveries and essential care for newborns is met, the need for management of complications is reduced, and all care required for complications is provided.

## Results

### All interventions – average direct costs

The average direct cost for providing each of the 109 interventions cost for AIU-2019 are provided in Supplementary Table 1. These are the basic financial requirements for offering care to each woman or newborn. Comparing modern contraceptive methods, the average annual cost per CYP, considering all 132 countries, was lowest for the IUD at US$0.93 per year, followed by male and female sterilisation (US$1.10 and 2.02, respectively). Although the cost of performing the sterilisation procedure or insertion and removal of the IUD may appear costly, when divided by the years of protection provided (10–13 for sterilisation and 4.6 for IUDs), the cost per CYP is very low when compared to other methods.

While average direct costs for all interventions are essential for budgeting SRH as part of UHC, a few specific services warrant further comment. STI treatment costs are very low (averaging less than US$7.50 per woman treated across all LMICs) due to the low-cost of the recommended antibiotics. However, the costs presented here do not include the treatment of drug-resistant strains, which are emerging globally due to over-use of antibiotics and poor disease management practices.

Use of mifepristone in safe MA regimens results in higher costs than for surgical safe abortion methods, due to the relatively high cost of mifepristone; however, the average cost of offering a safe MA is still lower than the average cost of offering PAC for severe complications from unsafe abortion. Considering all safe abortion methods – medical and surgical – in all LMICs, the average direct cost for providing safe abortion is roughly US$12. In comparison, the average direct cost of providing PAC for either shock, sepsis, uterine perforation or haemorrhage is roughly US$75, and providing PAC for all of these complications would cost an average of roughly US$300 per women served.

The most expensive services in the model are largely preventable. Looking across all LMICs, PAC for severe haemorrhage, caesarean sections (for obstetric complications or as elective procedures), and treatment of low birth-weight in newborns are among the top five most costly interventions. In contrast, services that prevent the bulk of the need for these services are less costly.

Supplementary Table 2 provides insight regarding the drivers of the total intervention costs. Looking across all LMICs, personnel costs are the largest cost category for 68% (74/109) of the interventions. This was despite following the WHO’s guidance for task-shifting to lower cadres of personnel when possible.^49^ Drug and supply costs follow, being most costly for 29 (27%) of the interventions. Those interventions generally included HIV treatment, interventions requiring blood products, newborn vaccinations, and supply-intensive interventions like management of newborn complications.

Costs are provided for five regions in Supplementary Table 2: Africa, Asia, East and Southern Europe, Latin America and the Caribbean, and Oceania. For contraceptive methods that required a commodity (i.e. excluding sterilisation and lactational amenorrhoea), commodity costs were highest in Latin America and the Caribbean for 50% (5/10) of the methods. Personnel costs were highest – for all interventions – in East and Southern Europe.

### Interlinked intervention examples – total direct costs

Table 4 provides the total cost, by region, of providing each of the 31 costed interventions included in the scenario analysis. The interventions for childbirth tend to be more costly than those for abortion, post-abortion care, and STI treatment. For all interventions except the less safe abortion MA procedures and newborn sepsis management, costs are highest in East and Southern Europe due to higher estimated personnel costs. For less safe medical abortions, the costs are assumed to be the same in all regions because they include the drug costs only.

**Table 4.** Total direct cost (USD) per recipient of selected interventions in LMICs in 2019, by region*

Intervention	Africa	Asia	East and Southern Europe	Latin America and the Carribean	Oceania	All LMICs
**Cluster 1: STI treatment for women aged 15–49**						
Chlamydia treatment	4.97	4.65	6.96	6.94	4.46	5.02
Gonorrhea treatment	4.93	4.61	6.92	6.89	4.42	4.98
Syphilis treatment	7.29	6.97	9.28	9.26	6.78	7.34
Trichomonas treatment	4.94	4.62	6.94	6.91	4.44	4.99
PID from chlamydia or gonorrhoea	7.09	6.78	9.09	9.06	6.59	7.15
**Cluster 2: Induced Abortion and Postabortion Care (PAC)**						
***Induced abortion services for safe abortions***						
Abortion - Manual or electric vacuum aspiration (safe)	4.69	4.42	6.36	6.19	4.26	4.72
Abortion - Dilation and evacuation (safe)	8.09	7.55	11.52	11.22	7.23	8.16
Abortion - Mifepristone and misoprostol (<12 weeks or 84 days) (safe)	19.19	18.76	21.88	21.28	18.50	19.20
Abortion - Mifepristone and misoprostol (≥12 weeks) (safe)	24.94	24.13	30.05	29.23	23.65	25.01
Abortion - Misoprostol (<12 weeks) (safe)	6.48	6.05	9.18	8.94	5.80	6.53
Abortion - Misoprostol (≥12 weeks) (safe)	8.69	8.26	11.39	11.08	8.01	8.74
***Induced abortion services for less safe abortions***						
Abortion - Manual or electric vacuum aspiration (less safe)	4.86	4.58	6.62	6.59	4.41	4.90
Abortion - Dilation and currettage (less safe)	8.47	7.93	11.90	11.86	7.61	8.56
Abortion - Mifepristone and misoprostol (<12 weeks or 84 days) (less safe)	13.81	13.81	13.81	13.81	13.81	13.81
Abortion - Mifepristone and misoprostol (≥12 weeks) (less safe)	14.91	14.91	14.91	14.91	14.91	14.91
Abortion - Misoprostol (<12 weeks) (less safe)	1.10	1.10	1.10	1.10	1.10	1.10
Abortion - Misoprostol (≥12 weeks) (less safe)	3.31	3.31	3.31	3.31	3.31	3.31
***Least safe abortion (all gestations)***						
*No health systems costs included*	0.00	0.00	0.00	0.00	0.00	0.00
**Postabortion care (PAC) for induced abortion or miscarriage complications**						
***PAC for incomplete abortion and nonsevere bleeding***						
PAC - Treating Incomplete abortion with manual vacuum aspiration (MVA)	11.07	10.28	15.99	15.92	9.82	11.20
PAC - Treating incomplete abortion with misoprostol	15.61	14.62	21.86	21.77	14.03	15.78
PAC - Management of non-severe haemorrhage	6.21	6.19	6.36	6.36	6.17	6.22
***PAC for abortion complications needing comprehensive emergency obstetric care (EmOC)***						
PAC - Shock	40.03	38.41	49.44	49.36	37.51	40.20
PAC - Sepsis	77.34	74.83	91.57	91.48	73.46	77.56
PAC - Uterine perforation/cervical laceration	69.26	66.19	86.61	86.50	64.52	69.52
PAC - Severe hemorrhage	119.86	117.34	134.09	134.00	115.97	120.08
***Prereferral management of abortion complications***						
PAC - Prereferral management of abortion complications	28.91	27.46	38.07	37.94	26.60	29.16
**Cluster 3: Safe childbirth services**^†^						
***Maternal care***						
Obstetric fistula repair	82.45	76.87	114.84	114.57	73.77	83.04
Maternal sepsis case management	109.31	105.07	134.06	133.84	102.70	109.77
Postpartum hemorrhage (PPH) treatment	105.51	100.87	132.71	132.47	98.27	106.03
***Newborn care***						
Newborn local infections treatment	1.95	1.81	2.83	2.81	1.72	1.97
Newborn sepsis treatment with injectable antibiotics	27.97	25.88	38.30	38.34	24.86	27.96
Newborn sepsis treatment with full supportive care	87.20	81.26	121.75	121.45	77.96	87.84

LMIC, low- and middle-income country; CEmOC, comprehensive emergency management of obstetric complications; PID, pelvic inflammatory disease.

* These costs are the sum of the components found in Supplementary Table 2.

^†^ Several interventions contribute to reductions in need for the impacted services (listed here). These include in-facility delivery and provision of essential newborn care, but many others are also included, such as management of obstructed labour, etc. Because of the complex nature of the linkages between these interventions we have not provided preventive service costs here.

Table 5 provides the total costs for providing care to women and newborns in all LMICs, currently and in the two all-needs met scenarios. (Supplementary Table 4 presents the total costs by region.) The total cost of treating chlamydia, gonorrhoea and PID in LMICs, given current care levels, is US$188.3 million annually. This includes US$127.3 million for treating PID in the many women who have untreated chlamydia and gonorrhoea. However, many cases of PID still go untreated. If all PID cases received treatment, total costs for the service would rise to US$227.2 million annually, but, fortunately, these are preventable costs. If treatment were extended to all women in need of chlamydia and gonorrhoea treatment, including both symptomatic and asymptomatic women, all need for PID treatment resulting from untreated chlamydia and gonorrhoea would be eliminated. Total costs would still rise to US$466.3 million per year, which is 148% more than current expenditure, due to very low current levels of treatment. However, it is important to remember that the financial and economic costs – and individual- and societal-level impact – of the infertility and newborn complications resulting from untreated chlamydia, gonorrhoea, and PID are not included in this example.

**Table 5.** Total cost (000s) (USD) for providing care by intervention and within linked category in all LMICs in 2019, by scenario*

Scenario:	(1) Current service levels	(2) All-needs-met: Impacted service(s)	(3) All-needs-met: Preventive & impacted services
**STI treatment**			
Chlamydia treatment	29,293	29,293	298,972
Gonorrhoea treatment	31,738	31,738	167,352
PID treatment	127,364	227,188	0
***Total***	***188,395***	***288,219***	***466,324***
**Induced abortion and postabortion care**			
Safe abortion services	353,002	353,002	*642,872*
Less safe abortion services	172,888	172,888	0
Least safe abortion services	0	0	0
Postabortion care^†^	869,437	1,479,415	*119,355*
***Total***	***1,395,327***	***2,005,306***	***762,227***
**Safe childbirth services**			
**Preventive - all services^ǂ^**	-	-	-
**Impacted**			
Obstetric fistula repair^§^	1,431	-	*0*
Maternal sepsis case management^§^	315,300	-	*347,236*
Postpartum haemorrhage (PPH) treatment^§^	711,573	-	*841,409*
Newborn local infections treatment	18,846	-	*11,874*
Newborn sepsis treatment (injectable antibiotics + full supportive care)	313,075	-	*206,746*
***Total***	***1,360,226***	-	***1,407,265***

LMIC, low- and middle-income country; PID, pelvic inflammatory disease; PPH, post-partum haemorrhage.

* Regional outcomes can be found in Supplementary Table 4.

^†^ For abortion complications (i.e. not including PAC for miscarriage).

^ǂ^ Several interventions contribute to reductions in need for the impacted services. These include in-facility delivery and provision of essential newborn care, but many others are also included, such as management of obstructed labour, etc. Because of the complex nature of the linkages between these interventions, we have omitted providing costs for all the preventive services here.

^§^ These costs are only for women needing these interventions who have live births. Those needing these interventions who have a stillbirth are excluded here.

For abortion services, current costs for safe and less safe procedures total US$525.9 million annually across all LMICs. PAC for management of abortion-related complications costs an additional US$869.4 million, resulting in a total of US$1.4 billion annually for abortion and PAC services. Yet, not all need for PAC is met. Meeting all need for this service would increase the total cost for PAC to US$1.5 billion. However, if – hypothetically – all unsafe abortion could be provided under safe conditions, greatly reducing abortion-related complications, PAC costs would reduce to US$119.3 million. Providing safe abortion services to all women currently experiencing safe, less safe, or least safe procedures, would increase abortion service costs in all LMICs by US$117.0 million annually, for a total of US$642.9 million. However, because of the large cost savings from reduced PAC spending (just over US$750 million) that results from shifting to safe abortion services, overall, the combined cost to health systems in LMICs for providing abortion and PAC services would decrease to US$762.2 million (down US$633.1 million, or 45%, from current total costs). Also, as with STI services, it is important to remember that the costs noted here do not include the significant health and societal benefits that would result from averting maternal mortality and other negative impacts from unsafe abortion.

Finally, we turn to the third example, which focuses on providing quality, in-facility childbirth services, including essential care for women and their newborns, and providing treatment for a select group of delivery-related complications. We estimate that there are 127.4 million live births in LMICs annually and that 96.6 million women and newborns have in-facility deliveries with the required essential care (Table 3). At current service levels, many women need but do not receive care for obstetric fistula repair, maternal sepsis case management, and postpartum haemorrhage treatment. Likewise, many newborns who need care for infections or sepsis do not receive it. The total cost for managing all complications in this cluster under current conditions is US$1.36 billion annually. For the all-needs-met scenario, all women and newborns receive safe childbirth services as recommended. As a result, all cases of obstetric fistula are eliminated due to women receiving appropriate care for obstructed labour (Table 3). Other maternal and newborn complications are greatly reduced. However, in the all-needs-met scenario all complications also receive treatment, and the overall costs increase by 3% to US$1.41 billion annually. Again, it is important to remember that these increased investments likely avert other, longer-term negative health and financial impacts.

## Discussion

SRH encompasses a broad range of health needs and services. This analysis provides a closer look at the interconnectedness of just a subset of these services; however, important lessons can be gleaned for governments and other stakeholders working towards ensuring universal access to the full package of SRH care. First, a key point arising from this analysis is the importance of considering the preventive potential of many SRH services in terms of both their health impact and cost-saving benefits. We show that meeting all need for a particular service will result in increased costs for that service alone; however, this increased investment can reduce the costs of meeting need for other services as well as total costs within a cluster of interlinked services.

Second, this analysis demonstrates the importance of acknowledging that not all SRH service investments will result in cost savings. Understanding the interconnectedness of many SRH services – not only within the health sector, but to other sectors as well – is important for financial planning. However, commitments to ensuring universal access must acknowledge first and foremost the rights of individuals to access care. Meeting all need for some SRH services will simply require greater investment.

As countries take steps towards realisation of universal access to care, policymakers should be cognisant of methods for priority setting (i.e. prioritising health care services for inclusion in financing schemes) that allow for greater transparency and consideration of multiple criteria. Multi-criteria decision analysis (MCDA) is one example. MCDA allows for objective quantification of many types of information, including whether the service will contribute to fulfilling the country’s larger health and rights commitments under the UHC rubric.^50,51^ It also calls for input from individuals with various perspectives, such as health care providers, health economists, individuals responsible for health policy and planning, treasury/finance representatives, and patient advocates.

The AIU-2019 cost model and analysis, and by extension this analysis, provide valuable insight regarding the potential costs and impacts of offering a package of SRH services. However, the approach does have limitations. The costing approach is normative in all scenarios. We cannot accommodate the potentially higher or lower costs of offering care that might occur in countries currently using inefficient or substandard approaches for service delivery. The costs are also assumed to be constant. We do not allow for increasing marginal costs to accommodate individuals not served by traditional service delivery models. Reaching these groups would likely cost more. Likewise, we do not include diminishing marginal costs for economies of scale. We are not able to incorporate considerations of who pays for care.

This analysis is also limited in that we do not account for the additional health system investments that would be required to achieve the all-needs-met scenario. One strategy to mitigate this last limitation is including indirect costs, referred to as programmes and systems costs. This is done in the larger AIU-2019 model. These programmes and systems costs are meant to cover the costs of infrastructural and other quality and capital improvements as well as the corollary, supportive services needed to offer the services included in the estimates. We did not include programmes and systems costs, which are calculated in AIU-2019 using markup rates, in this analysis because they could obscure the direct cost estimates and be less helpful for budgeting purposes in individual countries.

We acknowledge that a lack of sensitivity analysis is also a limitation. Many of the inputs represent point estimates drawn from country-level studies, and some parameters were estimated using regional information, estimates from all LMICs, or advice from experts. We are unable to quantify the impact of potential over- or under-estimation of these inputs on the findings. However, for all three interlinked service clusters, if spending on the “preventive” service (i.e. treatment of chlamydia and gonorrhoea, provision of safe abortion, and provision of safe delivery and essential newborn care) increases to meet all need, the costs of providing the impacted services will decrease or be eliminated altogether. This is certain. The uncertainty lies in whether the combined cost of the preventive and impacted services will increase or decrease when all need is met for the preventive service, and that is largely impacted by the degree to which need for the preventive service is currently being met. Individual countries would need to assess their own situation before assuming the aggregate results presented here are directly applicable.

Finally, this analysis and the larger AIU-2019 analysis are limited from a UHC perspective in that certain historically neglected SRH services, such as violence against women, diagnosis and treatment of reproductive cancers, and services addressing the needs of individuals with diverse sexual orientation and gender identities and expressions, are not included. The larger AIU-2019 analysis includes over 100 SRH interventions; however, full access to the comprehensive set of SRH services as defined in the Guttmacher-*Lancet* Commission would require greater investment than is estimated.^10^ That said, the Commission also speaks to the lack of data and the complexity of estimating need and coverage for the many SRH interventions not included in AIU-2019.

Fortunately, other initiatives have also attempted to assess the costs and benefits of offering SRH services globally, furthering the case for SRH investment and addressing some of the limitations of AIU-2019 and this analysis. These include the Disease Control Priorities project,^52^ the WHO’s cost estimates of achieving the health-related SDGs,^53^ and Sheehan et al’s case for global investment in adolescents,^54^ all of which highlight the benefits of multi-sectoral investment. The Reproductive Health Supplies Coalition’s Commodity Gap Analysis addresses the critical role of the private sector in contributing to increased access to services,^55^ and the Guttmacher-Lancet Commission summarises available evidence on the cost-effectiveness of SRH service provision.^10^ Taken together with AIU-2019 and this analysis, this collection of work provides ample guidance for policymakers aiming to explore the potential costs and impacts of investing in a wide range of SRH services, including for historically neglected groups, such as adolescents, and stigmatised services, such as safe abortion.

In the future, the global community and in-country policymakers alike could benefit from greater sharing of locally collected data on the costs and benefits of investing in SRH services, in public and private health sectors. Capacity to conduct costing is often limited, as is the funding to support it; however, governments and funders should consider the benefits of greater availability of up-to-date data for informing budgeting and planning for UHC. This analysis also shows that human resources comprise the largest category of direct SRH service costs, and underscores the need for continued investment in the training and retention of health care workers as countries strive to meet their UHC goals.

Countries committed to “health for all” have taken diverse pathways as they have progressed toward their goals of achieving UHC; yet there are overarching similarities: Political commitment to greater equity in health access, increased resources for health, and an increase in the share of resources that come from pooled sources are all important.^1^ In addition, data and systems for budgeting are critical.^11^ Looking forward, there is growing recognition that, in order to achieve country-level goals for health services delivery, consideration of commitments to human rights and social goals must be integrated into priority-setting processes, moving beyond considerations of costs alone.^50^ This is particularly important for SRHR, where, although commitments have been made, many individuals still face significant challenges in exercising their rights to access care. Addressing these challenges and facilitating full access to a comprehensive package of SRH services has the potential to offer society-wide benefits, leading to developmental opportunities in the longer term.^10^

## Supplementary Material

Supplementary Table 3

Supplementary Table 4

Supplementary Table 1

Supplementary Table 2

## Data Availability

The data that support the findings of this study are available at https://gu.tt/AddingItUp. The survey data used in the analysis include the Demographic and Health Surveys (DHS); Multiple Indicator Cluster Surveys (MICS); U.S. Centers for Disease Control and Prevention Reproductive Health Surveys (RHS); Performance Monitoring for Action (PMA) surveys; and Pan Arab Project for Family Health (PAPFAM) surveys. Restrictions apply to the availability of these data, which were used under license for the current study, and so are not publicly available. Data are available for free from many of the source websites (e.g. the DHS Program, UNICEF and PMA websites), respectively, or by request from country statistical offices.
